# Assessment of prescription completeness and drug use pattern using WHO prescribing indicators in private community pharmacies in Addis Ababa: a cross-sectional study

**DOI:** 10.1186/s40545-023-00607-3

**Published:** 2023-10-20

**Authors:** Yeniewa Kerie Anagaw, Liknaw Workie Limenh, Derso Teju Geremew, Minichil Chanie Worku, Misganaw Gashaw Dessie, Tewodros Ayalew Tessema, Teshome Bitew Demelash, Wondim Ayenew

**Affiliations:** 1 Department of Pharmaceutical Chemistry, School of Pharmacy, College of Medicine and Health Sciences, University of Gondar, Gondar, Ethiopia; 2 Department of Pharmaceutics, School of Pharmacy, College of Medicine and Health Sciences, University of Gondar, Gondar, Ethiopia; 3 Department of Pharmacy, College of Health Sciences, Debre Markos University, Debre Markos, Ethiopia; 4 Department of Pharmacy, Pawi Health Sciences College, Pawi, Ethiopia; 5 Department of Social and Administrative Pharmacy, School of Pharmacy, College of Medicine and Health Sciences, University of Gondar, Gondar, Ethiopia

**Keywords:** Essential medicines list, Prescribing pattern, Rational drug use, WHO core drug use indicators, Ethiopia

## Abstract

**Background:**

Healthcare systems in both developing and developed countries were not free from prescription errors. One of the effects of prescription errors is irrational prescribing. According to the estimation of the World Health Organization (WHO), greater than 50% of medicines are prescribed and dispensed irrationally. On the other hand, research on drug use patterns in the private healthcare sector is scarce. This study aimed to assess prescription Completeness and Drug use Pattern using WHO prescribing indicators in Private Community Pharmacies in Lemi-Kura sub-city.

**Methods:**

Based on the WHO prescribing indicators, a retrospective cross-sectional technique was employed to examine the completeness and drug-prescription patterns. The study was conducted from April to May 2021. Prescriptions, kept for 1 year that was prescribed from March 2020 to March 2021, by private healthcare sectors, were analyzed. A systematic random sampling technique was employed to select prescriptions obtained from private health facilities. Data were analyzed using SPSS^®^ version 26.0 software.

**Results:**

Of a total of 1000 prescriptions, 1770 drugs were prescribed and the average number of drugs per prescription was 1.77. Prescriptions for two drugs account for 38% of these, while prescriptions for three drugs account for 15%. Age, sex, and card number were written on 99.0%, 99.2%, and 41.8% of prescriptions, respectively. The patient's name was written on every prescription. Even though the availability of other therapeutic information on the prescription made it appear greater, only 44.2% of prescriptions included the dosage form of medications. The generic name was used for the majority of the medications (67.8%). Furthermore, assuming that each prescription was for a single patient, 71% of patients received antibiotics, and 2% received injectable medicines. The National List of Essential Medicines-Ethiopia was used in 99.6% of the prescriptions.

**Conclusions:**

On the basis of the finding of this study, the prescribing and prescription completeness indicator showed deviation from the standard recommended by WHO. This situation could be critical since a similar pattern is reported from public healthcare sectors, which might imply the extent of non-adherence to WHO core drug use standards. Consequently, it could play a considerable role in increasing prescription errors in Ethiopia. Hence, in-service training for prescribers should be provided to improve adherence to basic prescription writing.

## Background

Medicines are the key component of healthcare service provision [1]. The rational use of drugs is defined by World Health Organization (WHO) as “Patients get the best possible treatment at the affordable cost to them, in the best possible dosages, for an adequate period” [2]. Improvement of a patient illness condition is highly dependent on the rational use of drugs. However, the medication error could bring unnecessary impacts (such as adverse drug reactions and poor health delivery) on the patient [1].

The prescriber and dispenser are usually communicated by an instruction called a prescription [2]. The prescription could be considered a skill since it is the provision of instruction by the prescriber to the dispenser. The prescriber can be a doctor, a health officer, a midwife, a nurse, or a paramedical worker. Likewise, a dispenser might be a pharmacist, a pharmacy technician, or a nurse. Thus, by the principle, the prescription should be clear and legible and should indicate the precise item to be dispensed [3]. Prescribers regularly prescribe drugs. They are expected to apply their knowledge of therapeutics to select the right drugs for the right patients, and to prescribe the right doses for the right duration so as to maximize the patient benefit. However, inappropriate prescription of drugs that might be misused, and over or under-usage of medications could result in health hazards to the individual and community [4]. WHO and the International Network for Rational Use of Drugs (INRUD) stated some basic components of prescription should be indicated during prescription using a standard indicator [5–7]. A complete prescription should include the following information: the health facility level, the patient-related information (such as name, address, sex, age, and diagnosis), the drug’s name, strength, dosage form, frequency, and duration of treatment, as well as the prescriber and dispenser’s names and signatures [8].

Globally, the medication error is still a challenge [5–7]. According to the estimation of WHO, greater than 50% of medicines are prescribed and dispensed in an undesired way. Nearly half of the patients were not taking medicines correctly. Furthermore, nearly one-third of the world's population was unable to access essential medicines [5]. Both developing and developed countries health care systems were not free from inappropriate, futile, and economically ineffective use of drugs. These irrational medical practices were costing a lot of money in terms of negative clinical outcomes [2].

The prescription indicator will take a concerted effort to restructure pharmaceutical activities and practices to achieve reasonable medicine use. As a result, the WHO has developed a system that uses common indicators to track medicine use [7, 9]. Using prescription indicators aimed to follow up and assess the status of medication provided to the public. Assessing prescription indicators has several advantages in monitoring rational drug use. For instance, indicator helps health institutions to evaluate the trend of pharmacotherapeutic intervention taken in some sort of time [5]. It could also allow for enabling subsequent comparison of parameters between them, evaluate the population’s medication needs, and determine the most frequently used medications in a given area [9]. Moreover, the quality of service and organized information regarding the prescription profile could be assessed easily by the researchers [3].

Here are three important core drug use indicators, namely, prescribing indicators, patient care indicators, and facility indicators. For this study, we have used prescribing indicators. The commonly used WHO prescription indicators are the average number of drugs per medical prescription, percentage of drugs prescribed by generic name, percentage of drugs prescribed from essential drug list or formulary, percentage of encounters with an antibiotic prescribed, and percentage of prescribed injectable drugs [9]. The study aimed to identify the defects in prescription patterns to create awareness regarding the medication errors by giving feedback to the prescribers.

## Materials and methods

### Study area and period

The study was conducted at Lemi-Kura sub-city, Addis Ababa. Addis Ababa is the capital city of Ethiopia and has 11 administrative zones [10]. The sub-city of Lemi-Kura is east of the Addis Ababa municipal administration. It is a newly reorganized administrative zone composed of ten woredas, which has been active since 2020 [11]. This sub-city is populated with a variety of ethnic groups. In addition, not only Ethiopians but also most Eritreans live in this sub-city. The research took place between April and May 2021.

### Study design

A retrospective cross-sectional study design was carried out in ten private community pharmacies. We were used filed prescriptions from March 2020 to March 2021. All two groups of indicators (prescribing and prescription completeness indicators) were assessed based on the WHO guidelines [7]. Prescriptions prescribed by private health sectors for any diseases, with the exception of pregnant and psychiatric patients, were included in the study. Prescriptions from public healthcare facilities, prescriptions that were illegible or unclear, and prescriptions that were issued before or after the study time, were excluded from the study. All prescriptions dispensed from ten private community pharmacies placed at Lemi-Kura sub-city that was prescribed by private health institutions were taken as a source population, and those prescriptions dispensed within the study time frame were taken as study populations.

### Data collection and analysis

The principal investigators trained data collectors about the aim, methodology and data collection procedures. The data collection checklist was pretested at five private community pharmacies before the actual data collection and any necessary modifications were made. On a daily basis, data were cleaned to remove discrepancies and missing numbers. Three well-trained undergraduate pharmacy students were involved to collect data. They collected data from prescriptions and prescription registration books using a checklist. First, the prescriptions prescribed by the private health sector were chosen. Then after, a systematic random sampling technique was employed to select prescriptions obtained from private health facilities by taking every five prescriptions in pharmacies. Finally, we obtained one thousand prescription papers. The sample was taken from a 1-year prescription paper.

The data collection checklist mainly contains information about prescribers, patients, and drugs. Thus, patient information (age, sex, address, and full name), drug information (dosage form, strength, label, and total amount), and professionals’ information (name, address, telephone number of the prescriber, signature, or qualification of prescriber) were collected. Moreover, prescribing indicators include the average number of medicines per encounter, the percentage of medicines prescribed by generic name, the percentage of prescriptions with antibiotics, the percentage of prescriptions with injections, and the percentage of prescribed medicines from the essential medicines list (EML).

The data were entered and analyzed using SPSS V 26.0 software. In the statistical analysis, the required data to measure the prescribing indicators were recorded for each patient encounter and were entered directly into SPSS V.26.0. The indicator was reported as frequencies, averages/means, percentages, and proportions.

### Measurement tool for prescribing indicators

The WHO has developed and validated several indicators to provide an appropriate means to evaluate a nation’s medication use pattern and to measure the efficacy of interventions. The indicators are well-standardized, and they are suggested for use in drug use investigations. They provide a simple instrument for evaluating numerous essential aspects of pharmaceutical use in primary health care quickly and reliably. The final versions of the pretested indicators are described below [7].

The prescribing indicators that were measured include:

A. The average number of drugs prescribed per encounter was calculated by dividing the total number of different drug products prescribed by the number of encounters surveyed. Drug combinations prescribed for a single health issue were counted as one.
B. The percentage of encounters in which an antibiotic was prescribed was calculated by dividing the number of patient encounters in which an antibiotic was prescribed by the total number of encounters surveyed, multiplied by 100.
C. The percentage of encounters with an injection prescribed was calculated by dividing the number of patient encounters in which an injection was prescribed by the total number of encounters surveyed, multiplied by 100.
D. The percentage of drugs prescribed from an essential drug list (EDL) was calculated by dividing the number of products prescribed which are in the essential drug list by the total number of drugs prescribed, multiplied by 100.
E. The percentage of drugs prescribed by the generic name was calculated by dividing the number of drugs prescribed by the generic name by the total number of drugs prescribed, multiplied by 100.

### Operational definitions

Essential Medicines List: It is a collection of essential drugs or those that meet the population's most pressing healthcare needs. In this study, it was used interchangeably with the term „essential drug list [5].

Rational use of drugs: the word rational use means “prescribing the right drug with the right strength, for the right patient at the right dose for the sufficient time [5].

Prescribers’ adherence: it is the order writing in that prescribers had good adherence to the basic principles of prescription with some variables such as drugs per prescription, sex, age, generic name, card number, prescriber’s name, signature, strength and duration of drug and appropriate to the clinical needs of the patient at the lowest cost [13]. 

Antibiotics: These are substances produced by or derived from certain fungi, bacteria, and other organisms that can kill or impede the growth of other microbes. The term antibiotic is used as a synonym for medications used to treat bacterial infections in humans and animals in this study.

Combination of drugs: Two or more drugs for a specific health condition that are typically packaged, prescribed, and provided as a fixed-dose combination.

Generic name: This is the International Non-proprietary Name (INN) of a drug.

Prescription error: Prescribing that does not meet acceptable treatment standards. It includes poly-pharmacy, prescribing using proprietary brands or trade names of drugs, over-prescription of antibiotics and injections as well as prescribing too expensive drugs when cheaper equally effective alternatives are available.

## Results

### WHO core prescribing indicators

**Fig. 1:**
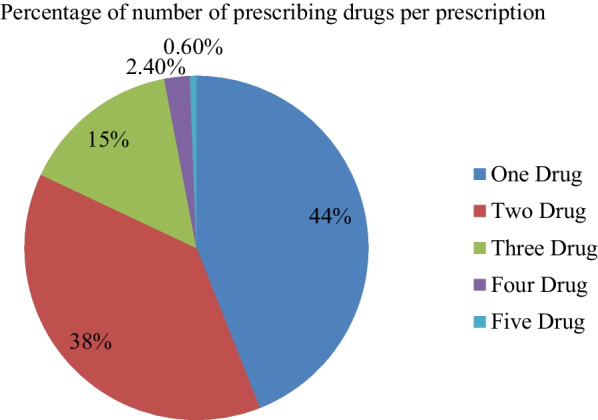
Percentage of the number of prescribing drugs per prescription among private community pharmacies (*N* = 1000 prescriptions)

Throughout this investigation, a total of 1770 medications were prescribed in 1000 prescriptions. The average number of drugs prescribed per encounter was found in WHO standard reference range (1.77) (Table 1). Out of all prescriptions, 560 (56%) of them had two and more drugs per prescription. While six prescriptions enclosed five drugs (Fig. 1). Only 678 (67.8%) prescriptions were prescribed by non-propriety names. Injections were prescribed in 18 (1.8%) of sampled prescriptions. Nearly 714 (71.0%) of prescriptions had at least one prescribed with antibiotics. Almost all 996 (99.6%) drugs were prescribed from the Ethiopian EML.

**Table 1:** WHO prescribing indicator in the prescription survey among private community pharmacies (*N* = 1000 prescriptions)

Indicators	*N* = 1000 (100%)	Standard values^5^
Average number of drugs per encounter (SD)	1.77 (0.8)	1.6–1.8
Percentage of drugs in generic name *N* (%)	678(67.8)	100
Percentage of injections *N* (%)	18(1.8)	13.4–24.1
Percentage of Antibiotics *N* (%)	714 (71.0)	20.0–26.8
Percentage of drugs from the Ethiopian essential medicine list (EML) or formulary *N* (%)	996 (99.6)	100

### Completeness of the prescription

This study evaluated the prescription completeness, i.e., patient information, treatment information, and professionals information. It was found that 99% and above of the prescriptions contain patient-related information (such as full name, age, and sex) except card number which was 418 (41.8%). Only 44.2% and 41% of prescriptions indicated the dosage form and diagnosis respectively even if the presence of other treatment information on the prescription seemed higher. On the professional-related information form, 28.8% of the prescribers put their qualification. Among them, 5% were Medical Doctors, 10% were Health officers, and 13.8% were Nurses. Moreover, professional information was very low, especially among pharmacists, where no dispensers were found to put their name and date of refill. While only 1.2% of druggists put their qualifications on the prescription papers (Table 2).

**Table 2:** Completeness of prescriptions among private community pharmacies (*N* = 1000 prescriptions)

Patient information parameters	*N* (%)	Treatment information parameters	*N* (%)
Full name	1000 (100)	Drug name, strength	994 (99.4)
Sex	992 (99.2)	Dose	906 (90.6)
Age	990 (99.0)	Frequency	982 (98.2)
Card number	418 (41.8)	Dosage form	442 (44.2)
		Diagnosis	410 (41.0)

Professional information			
Prescribers parameters	*N* (%)	Dispensers parameters	*N* (%)
Full name	334 (33.4)	Full name	86 (8.6)
Qualification	288 (28.8)	Qualification	12 (1.2)
Date	808 (80.8)	Date	9 (0.6)
Signature	964 (96.4)	Signature	116 (11.6)

## Discussion

The WHO core drug use indicators and prescription completeness were used in this study to assess prescription completeness and drug use indicators in private community pharmacies serving Lemi-Kura sub-city in Addis Ababa. The average number of drugs ordered per prescription in our study area is 1.77. This meets WHO prescribing indicator standard recommendations (1.6–1.8) [4]. In addition, this study is comparable with a study conducted in Tibebe-Ghion Comprehensive Specialized Hospital (1.65) [14]. However, some studies from Ethiopia reported the number of drugs per prescription is not comparable with our study and has not fallen under WHO's recommendation range. For instance, the study done in Hawassa showed 1.9 (SD = 0.91) drugs per prescription [12]. The average number of drugs per prescription was found to be 1.89 (SD = 1.16) in Tikur Anbesa Specialized Hospital (TASH) which is located in Addis Ababa [15]. Contrary to these, in a study conducted in southwest Ethiopia at Jimma Hospital, the average number of drugs per encounter was 1.59 [16]. In addition to these investigations, our finding is different from other studies done in Ethiopia revealed a higher number of drugs per prescription. For instance, 2.34 [17], 2.2 (SD = 0.8) [18], and 2.13 [19] per prescription were reported in the East, Northern, and south–west parts of Ethiopia, respectively. Likewise, a study was done in 12 developing countries on drug use patterns and the result was high in Nigeria (3.8) and low in Zimbabwe (1.3) [19, 20]. A high average number of drugs might be due to financial incentives to prescribers to prescribe more, lack of therapeutic training of prescribers, or shortage of therapeutically correct drugs. Low numbers could suggest a constraint of drugs or a lack of therapeutic abilities among prescribers [12].

Concerning the percentage of drugs prescribed by generic names, our study was (67.8%) below WHO prescribing indicator standard [4]. However, according to several studies done in Ethiopia, more than 90% of Ethiopian prescriptions contain generic medications. For instance, the percentage of drugs prescribed by the generic name was found to be 90.61% (85.04–92.26%) in public hospitals of eastern Ethiopia [17], (98.7%) in Hawassa [12], 97% in selected health facilities in eastern Ethiopia [18], respectively. Nonetheless, some studies done in Ethiopia reported the generic name of medicines was prescribed in less than 90% of prescriptions. Furthermore, some studies done in north–west [21] and Addis Ababa [22] revealed about 88% and 88.5% of medications were prescribed by generic name, respectively. The reason why it could be is the Ethiopian Pharmaceutical Supply Agency (EPSA), which procures generic medications, may be distributed to the public health sector based on the studies’ compliance with the requirements. However, in the present study, the minimal percentage of generic prescriptions from private health sectors could have several explanations. For example, they are not forced to purchase from public suppliers. Most health professionals and patients have misunderstood the safety and efficacy of generic medications. They have a choice to purchase from private importers which are more importing Brand products. There might be a limitation of generic drug distribution to the private health sector [23]. Most private health sector makes commercial partnership with brand medication importers. Another possible reason for reduced generic medication is the increased presence of several brands of medications in the market [12].

According to this study, the majority (99.6%) of medications in the private health sector are prescribed by the national EML. The current study was in comparison with similar studies done in public health sectors at Hawassa (96.6%) [12] and Bahir Dar (98.48%) [14]. The similarity of prescribing from both public and private could result in the better availability of essential drugs in the Ethiopian market so prescribers most likely prescribe those medicines.

The percentage of encounters in which antimicrobials were ordered in private health sectors was 71%, which is approximately 2.6 times more than WHO standard values (20–26.8%) [12]. This may be because of patients’ expectations, beliefs on antibiotics, and prescriber-related aspects. This finding is also in agreement with results (64.24%) from other studies [24]. The percentage of encounters in which injections were prescribed in the present study was 1.8%, which is significantly lower than the WHO requirement (13.4–24.1%) [12]. Professional and patient-related intervention activities may be the contributing factors. Our findings, however, were consistent with those of other relevant studies done in Mota (5.45%) and FinoteSelam (2.03%) [24].

In this study full name, age, sex, and card number of patients were mentioned in 100.0%, 99.0%, 99.2%, and 41.8% of prescription paper, respectively. Age and sex were well-included in studies done in South–West Ethiopia, which revealed that age, sex, and card number were mentioned in 81.8%, 76.3%, and 39.8% of the encounter, respectively [19]. The study conducted in Tibebe–Ghion Comprehensive Specialized Hospital showed good adherence with full name (100.0%), age (98.8%), sex (99.4%), and card number (99.1%) [14]. Likewise, research done in University Teaching Hospital shows good adherence with only full name (94.5%) but poor adherence with age (25.1%), sex (26%), and card number (22.4%). It implies that the majority of prescribers use the name of the patient to prescribe a drug than a card number [15]. The observed difference between different health facilities might be associated with the flow of customers making them inclined what easy to deliver the service. However, in only 44.2% of prescriptions, the type of dosage form was mentioned and extremely different from Bhosale et al. findings (77.93%) [25]. Even if it has to be 100%, about 96.2% and 98.2% of prescriptions had the duration and frequency of treatment in the present study, respectively. These findings were again higher than what was reported elsewhere [12, 26].

There were only 33.4%, 28.8%, 80.8%, and 96.4% of prescribers, who wrote their name, qualifications, and date, and put their signature on the prescription respectively, to assure they took responsibility for any accountability. In terms of this prescriber’s information, it was higher than Indian studies on qualification (21.75%) and signature (73.25%) [25]. However, this study's Professional information is lower than in the Nigerian study on prescriber names (95%) and signatures (98.2%) [26]. The poor prescriber information makes it difficult to identify the responsible prescriber for any feedback and clarification when required. It is extremely difficult to get prescription papers that carry the signature of the dispenser and only 11.6% put his/her signature on the prescription after dispensing drug(s) to the clients. Contrary to this, in the pediatric emergency ward of a tertiary hospital in Lagos, Nigeria, the dispensers put their signature on 92.1% of prescriptions after a refill [26]. Preparation and implementation of standard prescriptions in all departments and units of the health sector are crucial as there was a difference in the type and content of the prescriptions used by the practitioner. Training has to be provided to health professionals on good prescribing and dispensing practices to promote the rational use of drugs.

## Conclusion

Prescribing indicators in this study did not fulfill WHO recommended standard values of drugs per encounter. Prescribers had good adherence with some variables such as the number of drugs per prescription, sex, and age but poor adherence with some variables such as card number, prescribers name and signature, and strength of medications. In-service training should be provided to improve prescribers’ adherence to basic prescription writing.

## Data Availability

All data generated or analyzed during this study are included in this published article.

## Abbreviations

EDL: Essential drugs list
EFDA: Ethiopian Food and Drug Administration
EPSA: Ethiopian Pharmaceutical Supply Agency
FMOH: Federal Ministry of Health
SPSS: Statistical Package for the Social Sciences
TASH: Tikur Anbesa Specialized Hospital
WHO: World Health Organization
